# Liraglutide regulate osteogenesis-resorption via macrophage under compressive stress

**DOI:** 10.3389/fphar.2026.1985291

**Published:** 2026-10-26

**Authors:** Runze Kong, Difei Wu, Xiaochen Men, Yuzhu He, Qitao Du, Shuang Liu

**Affiliations:** 1 Stomatological Hospital of Dalian University (Dalian Stomatological Hospital), Dalian, China; 2 School of Stomatology, Dalian Medical University, Dalian, China; 3 Affiliated Zhongshan Hospital of Dalian University, Dalian, China

**Keywords:** compressive pressure, inflammation, liraglutide, osteoclastogenesis, osteogenic differentiation

## Abstract

**Objectives:**

Joint diseases always caused by mechanical pressure induced-inflammation and bone resorption. With advancing age, joint wear-and-tear intensifies while reparative capacity diminishes. Liraglutide is a common medication used to lower blood sugar and improve blood lipids, and it is widely used in elderly patients. This study investigated whether liraglutide (Lira) regulates macrophage-mediated bone metabolism under mechanical pressure and elucidated the molecular mechanisms.

**Methods:**

RAW264.7 macrophages were exposed to varying pressures (1–3 g/cm^2^) via custom-weighted glass coverslips. Cell viability was assessed by CCK-8 and Live/Dead staining. Cells were pretreated with 250 nmol/L Lira, and the expression of osteoclast-related (Piezo1, NFATc1, TRAP) and inflammation-related (TNF-α, iNOS, IL-1β, TRAF6, NF-κB/p-NF-κB) molecules was detected by qRT-PCR, immunofluorescence, and Western blotting. Conditioned media from treated macrophages were used to culture MC3T3-E1 pre-osteoblasts, and osteogenic markers (BMP2, RUNX2, ALP, OCN) were evaluated.

**Results:**

Pressure at 2 g/cm^2^ for 4 h optimally induced osteoclastogenesis and inflammation. Lira did not affect Piezo1 expression but significantly suppressed NFATc1 and TRAP at both mRNA and protein levels. Lira also inhibited the TRAF6-NF-κB pathway, reducing TNF-α, iNOS, and IL-1β expression. Conditioned media from Lira-treated macrophages significantly upregulated BMP2, RUNX2, ALP, and OCN in MC3T3-E1 cells, indicating indirect promotion of osteogenesis.

**Conclusion:**

Liraglutide reverses pressure-induced bone metabolic imbalance through a triple mechanism: inhibiting osteoclastogenesis, attenuating inflammation, and indirectly promoting osteogenesis via macrophage modulation. This study provides the first evidence of liraglutide’s molecular network in regulating bone metabolism under mechanical stress, supporting its therapeutic potential in pressure-related degenerative joint diseases such as osteoarthritis and temporomandibular joint osteoarthritis.

## Graphical Abstract

**Figure:**
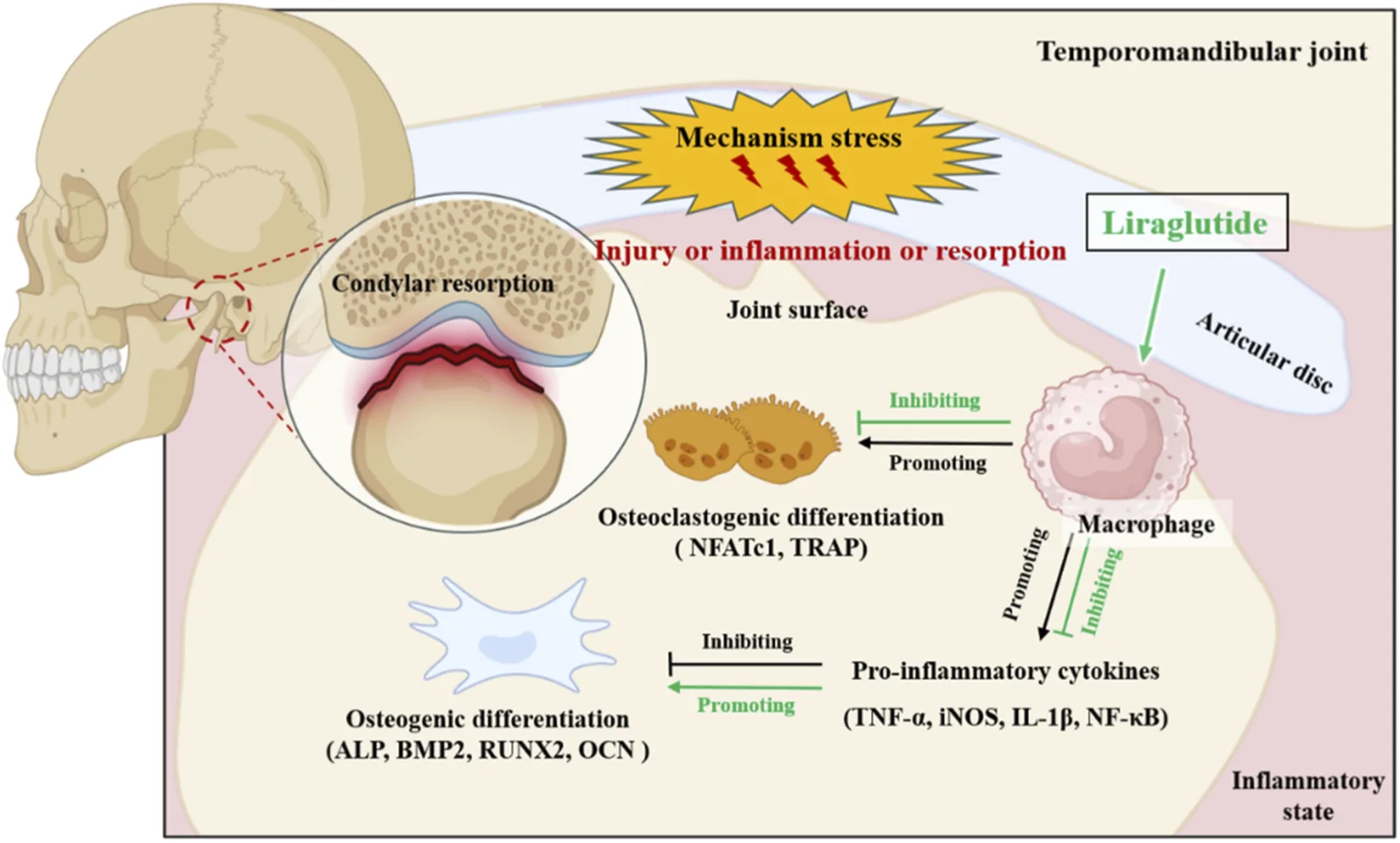

## 1 Introduction

Joints are the core structures of the human body responsible for weight-bearing, movement, and mechanical force transmission. Under physiological conditions, appropriate mechanical loading stimulates chondrocyte metabolism, promotes matrix synthesis, and maintains normal bone remodeling, thereby serving as a critical guarantee for joint tissue homeostasis (Blackler et al., 2025; Mertiya et al., 2023). However, prolonged overuse or internal structural disorders such as disc displacement expose joints to chronic abnormal mechanical stress, which disrupts joint homeostasis (Zhou et al., 2025); alternatively, diminished reparative capacity of the joint tissues may also contribute to this disruption. This initiates a cascade of pathological responses characterized by cartilage degeneration, synovial inflammation, and bone resorption, ultimately leading to degenerative joint diseases, among which osteoarthritis (OA) and temporomandibular joint osteoarthritis (TMJOA) are the most prevalent (Wang et al., 2025). OA is one of the leading causes of disability worldwide, with its prevalence continuously rising due to population aging. Meanwhile, TMJOA severely impairs patients’ quality of life by affecting essential physiological functions such as mastication and speech (Chen and Ren, 2026).

Glucagon-like peptide-1 receptor agonists (GLP-1RAs) have achieved remarkable success in the treatment of diabetes and obesity (Vilarrasa and Pellitero, 2025) and are widely used among elderly and obese populations. Notably, these two populations are precisely at high risk for joint inflammation, owing to physiological wear-and-tear and chronic excessive weight-bearing. Accumulating evidence suggests that liraglutide (Lira), a GLP-1RA, possesses direct bone-protective and anti-inflammatory properties, with effects that are, at least in part, independent of its glycemic or body weight regulatory functions (Gatto et al., 2025). At the cellular level, Lira has been shown to significantly inhibit RANKL- or LPS-induced osteoclast differentiation, accompanied by downregulation of key osteoclastic marker genes including NFATc1 and TRAP, and this effect is partially mediated through the suppression of NF-κB and MAPK signaling pathways (Li et al., 2020). In macrophages, Lira effectively inhibits polarization toward the pro-inflammatory M1 phenotype, reduces the release of inflammatory mediators such as TNF-α, and attenuates intracellular oxidative stress levels (Zhu et al., 2025). Furthermore, Lira has been demonstrated to promote osteoblast differentiation and mineralization, as evidenced by the upregulation of osteogenic markers including Runx2, ALP, and osteocalcin (OCN), suggesting its potential to facilitate bone formation (Zhai et al., 2023). The central focus of this study is to determine whether Lira can exert beneficial effects on joints concurrently with its glucose-lowering and weight-reducing actions.

Natural bone tissue possesses functional adaptability, enabling the activation of bidirectional regulatory pathways that promote osteogenesis and inhibit osteoclastogenesis in response to external mechanical stimuli, thereby achieving optimal structural and functional adaptation. However, chronic abnormal mechanical loading is one of the key initiators of joint degeneration. Under prolonged abnormal pressure loading, condylar chondrocytes and other joint-resident cells are activated to synthesize and secrete a plethora of inflammatory mediators, including NO, IL-1β, and MMPs (Tang et al., 2024; Zhang et al., 2025). Within this microenvironment, mechanical and inflammatory signals mutually reinforce each other, significantly amplifying the tissue-destructive effects. At the level of bone resorption, pressure overload can directly promote osteoclast generation and activation through specific signaling pathways. Concurrently, inflammatory responses also play a central role in joint bone resorption. Elevated levels of pro-inflammatory cytokines such as TNF-α and IL-1β are readily detected in the synovial fluid of patients with OA and TMJOA (Kaneyama et al., 2002). These inflammatory factors not only synergistically exacerbate cartilage matrix degradation but, more importantly, activate the NF-κB pathway in osteoblasts and synovial fibroblasts, leading to upregulated RANKL expression, which in turn enhances osteoclast differentiation and bone resorptive activity (Li et al., 2010; Rauner and Bozec, 2026). In this context, macrophages, as the most abundant immune cell population within joints, serve as an indispensable bridging element. Macrophages possess the ability to sense mechanical stimuli through various mechanosensitive channels (e.g., Piezo1) expressed on their surface, and they are also highly responsive to surrounding inflammatory cues (Saeki and Nakata, 2024; Zhu et al., 2026). Under the dual stimulation of mechanical and inflammatory signals, the polarization status and secretory function of macrophages undergo significant alterations, skewing toward a pro-inflammatory M1 phenotype and releasing large quantities of pro-osteoclastogenic factors. Through this mechanism, macrophages integrate mechanical and inflammatory signals and translate them into the final effector outcome of bone resorption.

In summary, joint abnormal pressure and inflammation synergistically drive bone resorption through complex interactive mechanisms, with macrophages serving as a core intermediary linking mechanical signaling, inflammatory responses, and bone metabolism. Given the combined anti-osteoclastogenic, anti-inflammatory, and osteogenic-promoting properties of Lira, a systematic investigation of its specific regulatory effects on macrophage differentiation and function under pressure-loaded conditions will not only deepen our understanding of the pathogenic mechanisms underlying degenerative joint diseases but also provide a critical theoretical foundation and experimental basis for the development of novel multi-target intervention strategies.

## 2 Materials and methods

### 2.1 Preparation of liraglutide

Liraglutide (A16115, Adooq Bioscience, United States) with a molecular weight of 3,751.202 Da was used in this study. The commercially available product contained 2 mg of liraglutide per vial, which was dissolved in 1 mL of ultrapure water to obtain a stock solution of 533.16 M × 10^−6^ M. This stock solution was subsequently diluted to a storage concentration of 250 μM, aliquoted, and stored at −20 °C. For each experiment, liraglutide was freshly diluted to the desired concentration with cell culture medium immediately before use.

### 2.2 Cell culture of RAW264.7

Murine macrophage RAW264.7 cells were cultured in complete medium consisting of DMEM (HyClone, United States) supplemented with 10% fetal bovine serum (FBS, Gibco, United States) and 1% penicillin-streptomycin (HyClone, United States). A cryovial containing RAW264.7 cells was retrieved from liquid nitrogen and rapidly thawed, and the cell suspension was transferred to the T25 flask. Cells were incubated at 37 °C in a humidified atmosphere containing 5% CO_2_, and cell growth was monitored every 12 h under an inverted microscope. When cells reached approximately 70% confluence, they were passaged for subsequent experiments. The experimental groups were designated as follows: Control group: RAW264.7 cells cultured in complete medium without any treatment; Press group: RAW264.7 cells cultured in complete medium and subjected to static pressure loading for 1, 2, 3, 4, 6, or 12 h; Press + Lira group: RAW264.7 cells cultured in complete medium and pretreated with 250 nmol/L liraglutide for 4 h prior to pressure loading.

### 2.3 Establishment and evaluation of the static pressure model

Establishment of the static pressure model in RAW264.7 cells: A gravity-loading static pressure device was employed in this study. Briefly, RAW264.7 were seeded at 2 × 10^5^/well in 6-well plates and 10^5^/well in 12-well plates. After cell adherence, specially customized smooth glass discs, which had been autoclaved, were placed on top of the monolayer cells. For 6-well plates, the glass discs had a diameter of 30 mm, a density of 2.5 kg/m^3^ × 10^3^ kg/m^3^, and thicknesses of 4, 8, or 12 mm. For 12-well plates, the glass discs had a diameter of 22 mm, a density of 2.5 kg/m^3^ × 10^3^ kg/m^3^, and thicknesses of 4, 8, or 12 mm. The pressure exerted on the cells was calculated according to formula P = ρh (where P represents pressure, ρ is the density of the glass, and h is the thickness of the glass disc), yielding pressures of 1, 2, and 3 g/cm^2^, respectively. The plates containing the glass discs were returned to the CO_2_ incubator for 4 h of culture. After the incubation period, the glass discs were carefully removed, and cells were collected for subsequent assays.

### 2.4 Establishment of the indirect co-culture model with MC3T3-E1 cells

The RAW264.7 cells were treated according to the method described in Section 2.3. After treatment with pressure and/or Lira, the culture supernatant was collected and mixed with α-MEM at a 1:1 ratio (v/v) to prepare conditioned medium for indirect culture of MC3T3-E1 cells. MC3T3-E1 cells were seeded at 5 × 10^4^ cells/cm^2^ in 24-well plates for alkaline phosphatase (ALP), and at 10^5^ cells/well in 6-well plates for RT-qPCR and Western blotting(WB) in conditioned medium for 24 h, after which the medium was replaced with osteogenic induction medium (α-MEM medium containing 10% FBS, 1% PS, 10 mM β-glycerophosphate, 50 μg/mL ascorbic acid, and 10 nM dexamethasone) for subsequent experiments.

### 2.5 CCK-8 assay for screening optimal pressure conditions

The RAW264.7 cells were seeded in 12-well plates at a density of 10^5^ cells per well. After 24 h of incubation to allow cell attachment, the glass discs were placed on the cells as described above. Cells were cultured for 1, 2, 3, 4, 6, or 12 h at 37 °C. Following the treatment, cells were washed with PBS, and 300 µL of CCK-8 solution (Abbkine, United States) was added to each well. The plates were incubated at 37 °C in the dark for 2 h. Subsequently, 100 µL of the supernatant was transferred to a 96-well plate, avoiding bubble formation, and the absorbance at 450 nm was measured using a microplate reader to determine the optical density (OD) values. All experiments were repeated independently at least three times (n ≥ 3).

### 2.6 Live/dead staining for evaluating pressure parameters

Cells in the logarithmic growth phase were harvested and seeded into 12-well plates at a density of 1 × 10^5^ cells per well. After 24 h of incubation, the glass discs were placed on the cells as described above. Cells were cultured for 1, 2, 3, 4, 6, or 12 h at 37 °C. After treatment, cells were washed twice with PBS for 5 min each. Subsequently, 500 µL of Live/Dead staining solution was added to each well, and the plates were incubated at 37 °C in the dark for 30 min. Cells were then washed twice with PBS and observed under an inverted fluorescence microscope for image acquisition.

### 2.7 Quantitative real-time PCR (qRT-PCR)

After treatment according to the methods described in Section 2.3, total RNA was extracted from the cells, and RNA concentration was determined using a UV spectrophotometer (NanoDrop-1000). Reverse transcription was performed using the Evo M-MLV Reverse Transcription Kit Ver. 2 (AG, China) according to the manufacturer’s instructions. Quantitative real-time PCR was carried out on a CFX96 Real-Time System. The primer sequences used are listed in Table 1. All experiments were repeated independently at least three times (n ≥ 3).

**TABLE 1:** Primer sequences for qRT-PCR.

Gene	Forward primer (5′-3′)	Reverse primer (5′-3′)
GAPDH	TGT​GTC​CGT​CGT​GGA​TCT​GA	TTG​CTG​TTG​AAG​TCG​CAG​GAG
piezo1	GCA​GCC​GAG​AGA​CAG​AGA​AG	AGA​GCA​GAG​GGA​ACC​AGA​TGA
NFATc1	AGA​AGA​TGG​TGC​TGT​CTG​GC	TCC​CGG​TCA​GTC​TTT​GCT​TC
TNF-α	CAG​AAA​GCA​TGA​TCC​GCG​AC	CTG​CCA​CAA​GCA​GGA​ATG​AG
iNOS	ACG​AGA​CGG​ATA​GGC​AGA​GA	GCA​CAT​GCA​AGG​AAG​GGA​AC
IL-1β	TGG​TGT​GTG​ACG​TTC​CCA​TTA	TCG​TTG​CTT​GGT​TCT​CCT​TGT
NF-κB	AGG​CAA​GGA​ATA​ATG​CTG​TCC​TG	ATC​ATT​CTC​TAG​TGT​CTG​GTT​GG
TRAP	TGG​TCA​TTT​CTT​TGG​GGC​TTA​TCT	GCT​ACT​TGC​GGT​TTC​ACT​ATG​GA
ALP	TAC​ATT​CCC​CAT​GTG​ATG​GC	AGA​GCA​GAG​GGA​ACC​AGA​TGA
RUNX2	TCT​CCA​ACC​CAC​GAA​TGC​ACT​A	ATA​GCG​TGC​TGC​CAT​TCG​AGG​T
BMP-2	AAC​GAG​AAA​AGC​GTC​AAG​CC	AGG​TGC​CAC​GAT​CCA​GTC​AT
OCN	CAG​AAC​AGC​AAG​TCC​CAC​ACA​G	TCA​GCA​GAG​TGA​GCA​GAA​AGA​T

### 2.8 Immunofluorescence staining (IF)

As the methods described in Section 2.3, the MC3T3-E1 cells were treated with the conditional medium for 24 h followed by osteogenic induction medium for 7 days. Then the cells were washed with PBS and fixed with 4% paraformaldehyde (Coolaber, China) at room temperature. Cells were permeabilized with 0.3% PBST and blocked with goat serum for 1 h at room temperature. Primary antibodies against TRAF6 (1:200), NFATc1 (1:200), and NF-κB p65 (1:100) were diluted in antibody diluent and incubated with cells overnight at 4 °C (12–16 h). After washing with PBS, cells were incubated with fluorescence-conjugated secondary antibody Goat Anti-Rabbit IgG (Dylight 488, Abbkine, United States) for 1–2 h at room temperature in the dark. Finally, cells were mounted with DAPI solution containing anti-fade reagent (Abbkine, United States). Images were captured using a fluorescence microscope (Zeiss, Germany), and semi-quantitative analysis of fluorescence intensity was performed using ImageJ software. All experiments were repeated independently at least three times (n ≥ 3).

### 2.9 Alkaline phosphatase (ALP) and Alizarin Red S (ARS) staining

The MC3T3-E1cells cultured as the method in 2.4 were seeded in 24-well plates at a density of 5 × 10^4^ cells/cm^2^. After the cells reached 90%–100% confluence, MC3T3-E1 cells were treated as described in Section 2.4 and subjected to osteogenic induction for 7, 14, and 21 days. The cells were then fixed with 4% paraformaldehyde for 20 min. A staining solution was prepared by mixing 20 μL NBT, 10 μL BCIP, and 3 mL chromogenic buffer, and the cells were incubated in it at room temperature in the dark. After rinsing, the cells were observed and photographed under a microscope. For Alizarin Red staining, cells fixed as described above were stained with Alizarin Red solution, and a small amount of anhydrous ethanol was added to keep the cells moist before observation and photography under a microscope. Then, 300 μL of elution buffer was added to each well, and the eluted Alizarin Red S solution was collected and mixed thoroughly. A 100 μL aliquot was transferred to a 96-well plate, and the OD value was measured at 560 nm.

### 2.10 Western blotting (WB)

After treatment according to the methods described in Section 2.3, total protein was extracted using the Animal Cell/Tissue Total Protein Extraction Kit (Column Method) (KTP3007, Abbkine, United States) following the manufacturer’s instructions. Protein extraction filter cartridges were placed into collection tubes and pre-chilled on ice. Cells were scraped and collected, then centrifuged at 1,200 rpm for 5 min to obtain cell pellets. Protease Inhibitor Cocktail was added to Native Cell Lysis Buffer at a ratio of 1:100 (v/v), and the appropriate volume of this lysis buffer was added to each sample. The lysed cells were transferred to pre-chilled column cartridges and centrifuged at 14,000 × g. The collection tubes were placed on ice, and the filter cartridges were discarded, leaving the extracted protein in the collection tube.

Protein concentrations were determined using a protein assay kit (KTD3001, Abbkine, United States) according to the manufacturer’s instructions. A standard curve was constructed, and the equation was considered valid when R^2^ > 0.99, allowing calculation of sample protein concentrations.

One-quarter volume of 5× Loading Buffer was added to each protein sample, mixed thoroughly, and heated at 99 °C for 10 min in a metal bath, followed by storage at −20 °C.

Sodium dodecyl sulfate-polyacrylamide gel electrophoresis (SDS-PAGE) gels were prepared using a PAGE Gel Rapid Preparation Kit (Epizyme, China). After gel polymerization, electrophoresis buffer and transfer buffer were prepared. Protein samples were subjected to electrophoresis, transferred to PVDF membranes, blocked, and incubated with primary and secondary antibodies. Finally, the membranes were developed using chemiluminescent substrate, and images were analyzed using ImageJ software. All experiments were repeated independently at least three times (n ≥ 3).

### 2.11 Statistical analysis

Data are presented as mean ± standard deviation from at least three independent biological replicates. Multiple group comparisons were performed using one-way ANOVA followed by *post hoc* tests (LSD when variances were equal; a correction method when variances were unequal), after confirming normality (Shapiro–Wilk) and homogeneity of variance. Analyses were conducted using SPSS 25.0 and GraphPad Prism 9.0. Statistical significance was set at P < 0.05 (*) and P < 0.01 (**).

## 3 Results and discussion

### 3.1 The osteoclastogenic and inflammatory effects of pressure loading on macrophages

In this study, glass coverslips of varying weights were placed on macrophages to apply pressures of 1, 2, and 3 g/cm^2^. The CCK-8 assay revealed that cell viability decreased in a pressure-dependent manner at equivalent time points. When macrophages were exposed to different pressures for 0–12 h, cell viability was markedly reduced between 2 and 4 h; notably, under 2 g/cm^2^ at 3 h, cell viability dropped below 50% of the control level (Figure 1A). Live-dead staining results corroborated these findings (Figure 1B). This pressure-dependent reduction in viability suggests that macrophages are highly susceptible to mechanical overload, which may compromise their physiological functions and potentially limit their capacity to mount subsequent effector responses. The observed viability threshold is consistent with previous reports indicating that excessive mechanical stress induces cytoskeletal disruption and mitochondrial dysfunction in immune cells, thereby imposing a ceiling on the magnitude of pressure that can be applied in experimental settings (Schröder et al., 2020; Wang et al., 2020).

**FIGURE 1:**
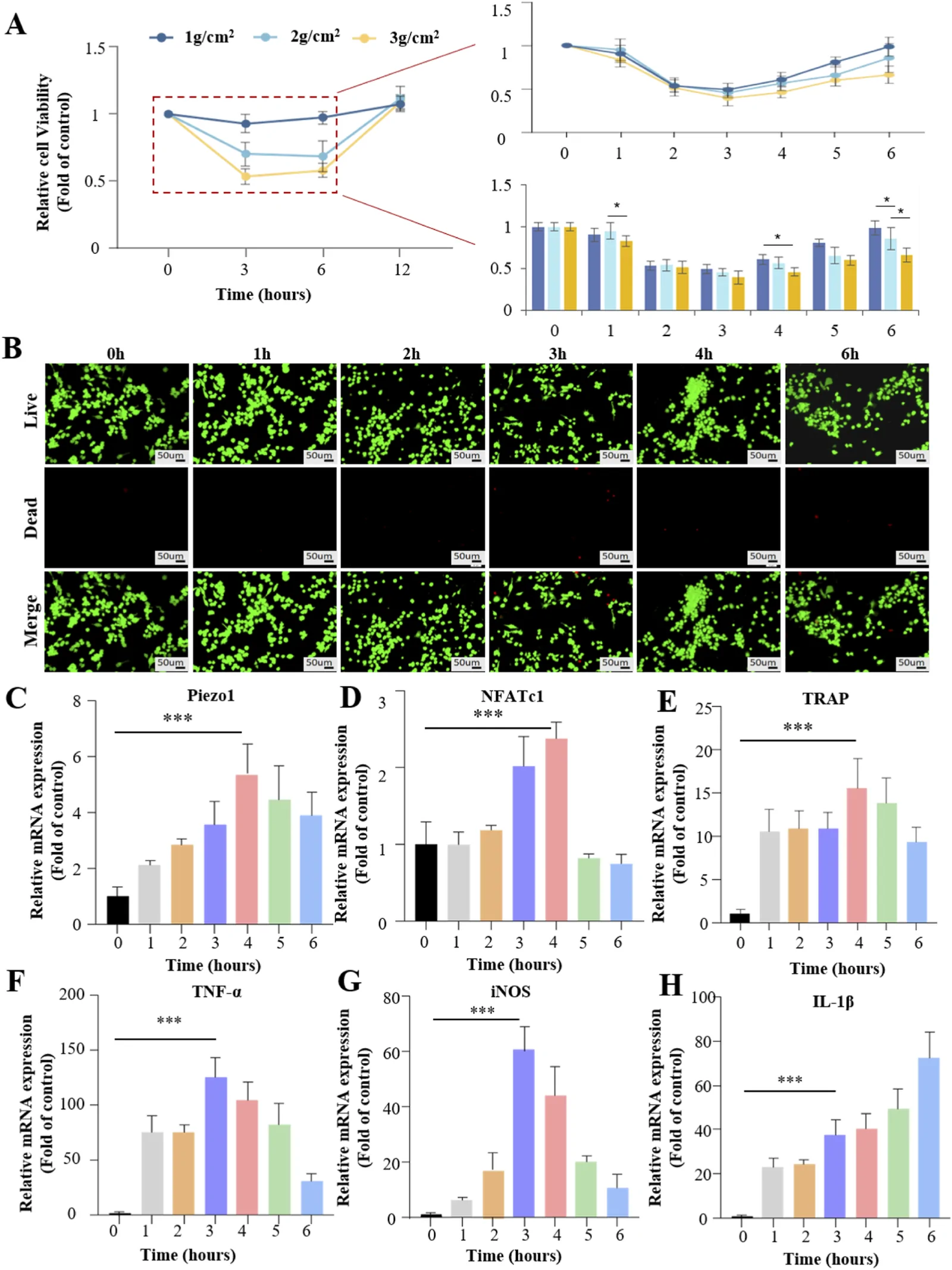
The effects of compressive pressure on macrophage viability and function treated by compressive pressure. **(A)** The viability of Raw264.7 from 0 to 12 h under pressures of 1, 2, and 3 g/cm^2^; **(B)** The live-dead staining of Raw264.7 under 2 g/cm^2^ pressure from 0 to 6 h; **(C–H)** The mRNA expression level of Piezo1 **(C)**, NFATc1 **(D)**, TRAP **(E)**, TNF-α **(F)**, iNOS **(G)**, IL-1β **(H)** in Raw264.7 under 2 g/cm^2^ pressure from 0 to 6 h (*P < 0.05 and ***P < 0.001 compared with the 0 h as control group).

Piezo1, a mechanosensitive ion channel located on the plasma membrane, serves as a pressure transducer that reflects the cellular response to mechanical stimulation. RT-PCR analysis showed that Piezo1 mRNA expression peaked at 4 h under 2 g/cm^2^ pressure (Figure 1C). Similarly, the mRNA levels of NFATc1 and TRAP were both maximally upregulated at the 4 h compared with 0 h (Figures 1D,E). NFATc1 is the master transcription factor governing osteoclast differentiation, whereas TRAP is a specific functional enzyme and a well-established histochemical marker for osteoclasts, providing direct evidence of osteoclast formation (Nugraha et al., 2023). The temporal coincidence of Piezo1, NFATc1, and TRAP peak expression strongly suggests that the mechanical signal transduced by Piezo1 converges upon the NFATc1 signaling axis to initiate the osteoclast differentiation program. Mechanical overload can directly promote osteoclast differentiation via Piezo1-mediated calcium influx and subsequent NFATc1 activation (Zhu et al., 2026; Zheng et al., 2026). Collectively, these data indicate that macrophages subjected to 2 g/cm^2^ pressure for 4 h exhibit a clear propensity toward osteoclastic differentiation. Although cell viability declined transiently under pressure, it recovered from 3 h onward, and live/dead staining confirmed that the vast majority of cells remained viable at 4 h. The peak induction of the mechanosensitive gene Piezo1 at 4 h, together with the coordinated and liraglutide-reversible changes in inflammatory and osteoclastic markers, indicates that the observed molecular changes reflect specific mechanotransduction rather than nonspecific cell injury.

Regarding the inflammatory response, TNF-α and iNOS expression levels were both significantly elevated at 3 and 4 h under 2 g/cm^2^ pressure compared with 0 h (Figures 1F,G), whereas IL-1β expression displayed a time-dependent increasing trend (Figure 1H). The concurrent upregulation of these pro-inflammatory mediators under the same pressure conditions underscores the intimate crosstalk between mechanical stress and inflammatory activation. It is conceivable that pressure-activated Piezo1 not only triggers the osteoclastogenic cascade but also instigates a local inflammatory milieu, characterized by the release of TNF-α, iNOS, and IL-1β, which may in turn potentiate NFATc1 activity and further amplify bone resorption. This dual effect—directly promoting osteoclast differentiation while simultaneously creating a pro-inflammatory microenvironment—likely recapitulates the pathophysiological processes underlying pressure-related degenerative joint diseases, such as osteoarthritis and temporomandibular joint osteoarthritis (Yu et al., 2025; Shibusaka et al., 2026). Based on these observations, a pressure of 2 g/cm^2^ applied for 4 h was selected as the optimal condition for all subsequent experiments, as this time point captured both the peak osteoclastogenic signaling and a robust inflammatory response, while maintaining sufficient cell viability for downstream functional assays.

### 3.2 Lira preventing pressure-induced osteoclastic differentiation of macrophages

Having established the optimal parameters for pressure treatment of macrophages in the preceding section, this part of the study was designed to investigate the effects of liraglutide (Lira) on pressure-induced osteoclastogenesis in macrophages (Figure 2A). RT-PCR analysis revealed that mechanical pressure significantly upregulated Piezo1 mRNA expression; however, Lira treatment did not exert any notable effect on Piezo1 transcript levels (Figure 2B). In contrast, Lira markedly suppressed the pressure-induced upregulation of NFATc1 and TRAP gene expression, two key molecular hallmarks of the osteoclastogenic pathway (Figures 2C,D). To corroborate these findings at the protein level, immunofluorescence staining and Western blotting were performed to detect NFATc1 and TRAP protein expression (Figures 2E,F). The results obtained from these protein-level assays were fully consistent with the gene expression data, further validating the inhibitory effect of Lira on the osteoclast differentiation program.

**FIGURE 2:**
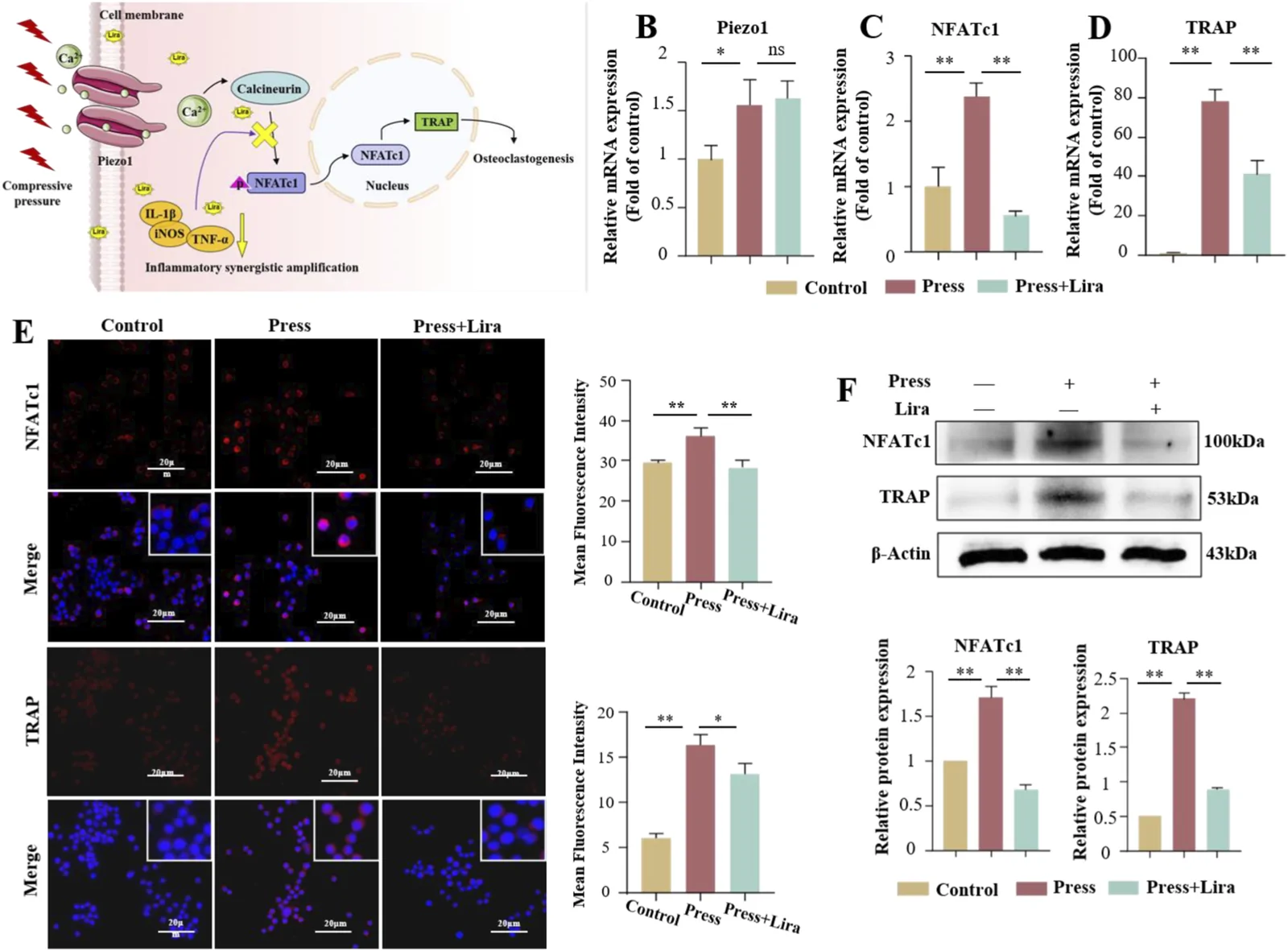
The effects of Lira on pressure-induced osteoclastogenesis in Raw264.7. **(A)** Schematic diagram of Lira on macrophage differentiation mechanisms under mechanical pressure; **(B–D)** The Piezo1 **(B)**, NFATc1 **(C)** and TRAP **(D)** mRNA expression levels in Raw264.7 cells under 2 g/cm^2^ for 4 h; **(E)** IF staining of NFATc1 and TRAP in Raw264.7 cells under 2 g/cm^2^ for 4 h; **(F)** NFATc1 and TRAP protein expression levels in Raw264.7 cells under 2 g/cm^2^ for 4 h (ns P ≥ 0.05, *P < 0.05, **P < 0.01).

Piezo1, as a mechanosensitive ion channel located on the plasma membrane, serves as the primary sensor for mechanical stimuli at the cellular level. The inability of Lira to alter Piezo1 expression suggests that its anti-osteoclastogenic action does not occur at the level of mechanosensing *per se*, but rather at downstream signaling nodes within the mechanical transduction cascade. The significant suppression of NFATc1 and TRAP expression by Lira, both at the mRNA and protein levels, points to a critical intervention point downstream of Piezo1 (Yu et al., 2021). The concurrent downregulation of both molecules under Lira treatment indicates that the drug effectively intercepts the mechanical signal after it has been transduced by Piezo1, thereby blocking the activation of the NFATc1-dependent transcriptional cascade. This finding aligns with previous reports demonstrating that GLP-1 receptor agonists, including Lira, can suppress osteoclastogenesis through inhibition of NF-κB and MAPK signaling pathways, both of which converge upon NFATc1 activation (Li et al., 2020; Zheng et al., 2025).

### 3.3 Lira suppressing pressure-induced inflammatory responses in macrophages

Given that inflammatory cytokines may exert synergistic amplification effects on macrophage osteoclastogenesis and also influence osteoblast differentiation, we next evaluated the effects of Lira on the inflammatory profile of macrophages under mechanical pressure. RT-PCR analysis revealed that Lira significantly suppressed the expression of the pro-inflammatory cytokines TNF-α, iNOS, and IL-1β (Figure 3A). To further investigate the underlying molecular mechanisms, immunofluorescence staining and Western blotting were performed to detect the expression of key molecules involved in the inflammatory signaling cascade. The results demonstrated that Lira markedly inhibited the protein expression levels of TRAF6, NF-κB (P65), and phosphorylated NF-κB (p-P65) (Figures 3B,C).

**FIGURE 3:**
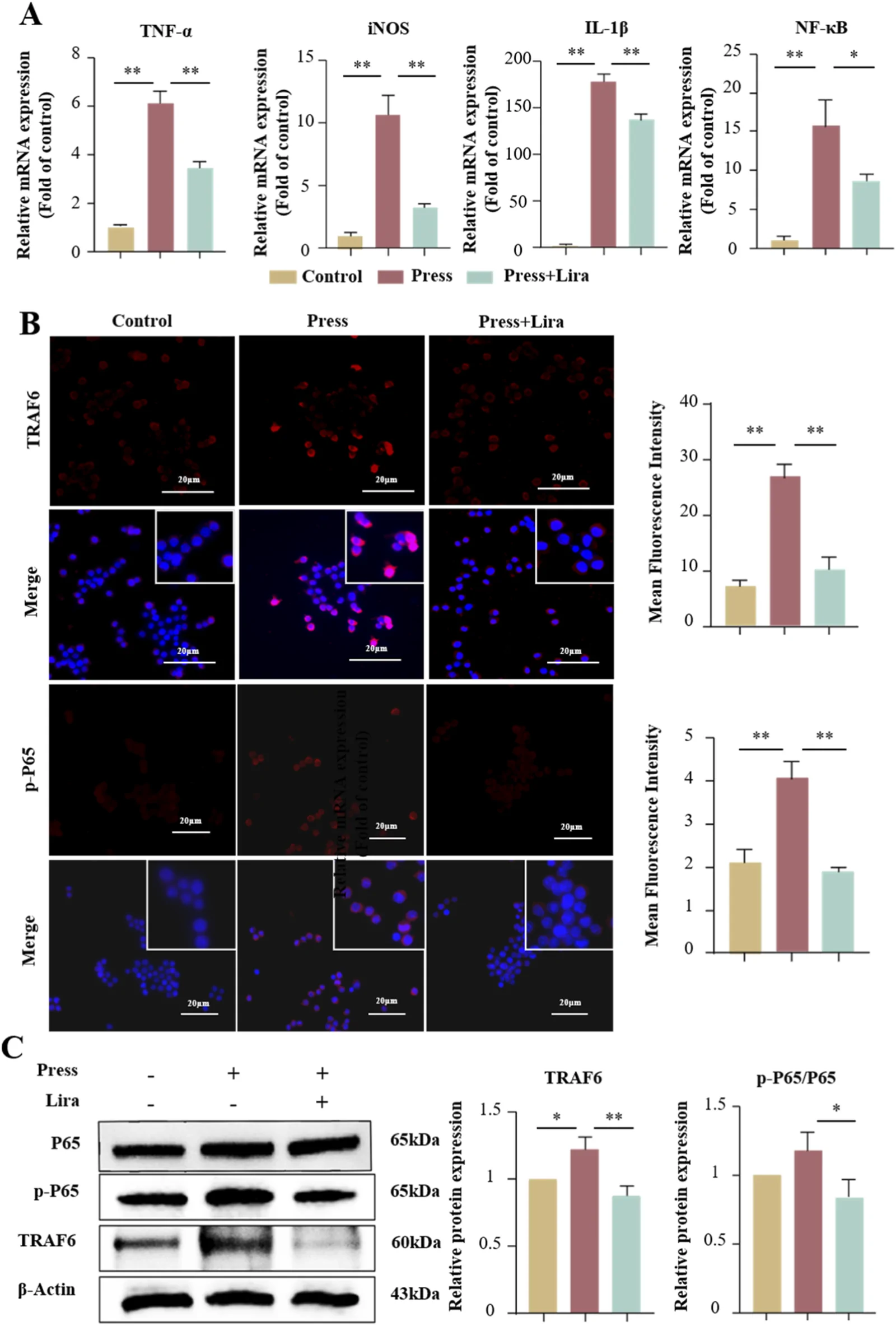
The effects of Lira on pressure-induced inflammation in Raw264.7. **(A)** The expression of inflammatory cytokines (TNF-α, iNOS, IL-1β and NF-κB) in Raw264.7 cells under 2 g/cm^2^ for 4 h; **(B)** The IF staining and fluorescence intensity of TRAF6 and p-P65 in Raw264.7 cells under 2 g/cm^2^ for 4 h; **(C)** The TRAF6, P65 and p-P65 protein expression levels in Raw264.7 cells under 2 g/cm^2^ for 4 h (ns P ≥ 0.05, *P < 0.05, **P < 0.01).

Mechanical stimulation itself can serve as an inflammatory trigger. TNF-α, iNOS, and IL-1β are well-established inflammatory mediators that not only amplify the inflammatory cascade but also synergize with osteoclastogenic signals to potentiate NFATc1 activity and promote bone resorption. Therefore, the ability of Lira to suppress these inflammatory factors suggests that its anti-osteoclastogenic effect may be partially attributed to the amelioration of the inflammatory microenvironment, which would otherwise exacerbate the pressure-induced osteoclast differentiation program. TRAF6 functions as a key adaptor molecule that bridges inflammatory receptors (such as TLRs and IL-1R) to the canonical NF-κB pathway. Upon activation, NF-κB undergoes phosphorylation and translocates to the nucleus, where it drives the transcription of a variety of pro-inflammatory genes, including TNF-α, iNOS, and IL-1β. The suppression of TRAF6, total NF-κB, and phosphorylated NF-κB by Lira indicates that the drug interferes with this signaling axis at multiple levels—both by reducing the abundance of the upstream adaptor and by directly attenuating the activation of the central transcription factor. This finding aligns with previous reports demonstrating that GLP-1 receptor agonists, including Lira, can inhibit the NF-κB signaling pathway, thereby exerting anti-inflammatory effects in various cell types (Wong and Drucker, 2025; Ngabea and Dimeji, 2025).

Collectively, these results indicate that Lira exerts a dual protective effect on macrophages under mechanical pressure: it directly inhibits the osteoclastogenic transcription factor NFATc1 and its downstream effector TRAP, while simultaneously suppressing the inflammatory milieu through inhibition of the TRAF6-NF-κB axis. This combined action likely disrupts the synergistic crosstalk between inflammatory signals and osteoclastogenic signals, thereby effectively counteracting pressure-induced bone resorption. These findings further support the therapeutic potential of Lira in pressure-related degenerative joint diseases, where mechanical overload and chronic inflammation jointly drive tissue destruction. However, whether liraglutide regulates macrophages in a GLP-1R-dependent manner still requires blocking or silencing GLP-1R to be definitively established.

### 3.4 Lira promoting macrophage-mediated osteoblast differentiation under compressive pressure

Metabolic imbalance in arthritis and other pressure-related bone disorders typically manifests as enhanced osteoclastic bone resorption accompanied by reduced bone formation. On this basis, we further investigated the effects of liraglutide (Lira) on macrophage-mediated regulation of osteogenic differentiation under mechanical pressure (Figure 4). Following culture of MC3T3-E1 cells in macrophage-conditioned medium for 7 and 14 days, RT-PCR analysis revealed that Lira significantly upregulated the mRNA expression levels of BMP2, RUNX2, ALP, and OCN in MC3T3-E1 cells (Figure 4A). Immunofluorescence staining results further demonstrated that Lira markedly enhanced the fluorescence intensities of ALP and RUNX2 in MC3T3-E1 cells under conditioned culture conditions, which were consistent with the gene expression findings (Figure 4B).

**FIGURE 4:**
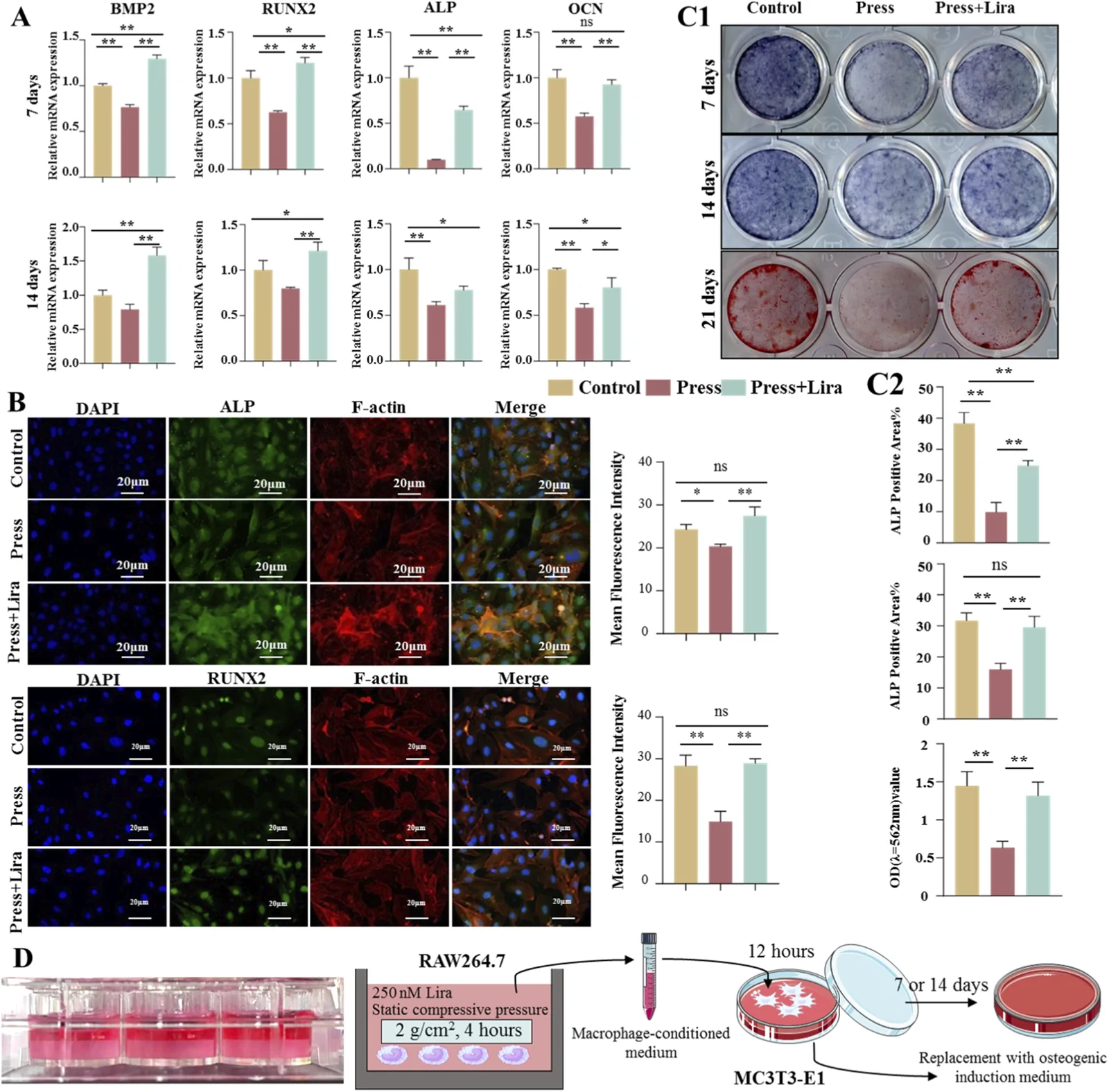
The effects of Lira on pressure-treated MC3T3-E1 differentiation via the Raw264.7 pressured with 2 g/cm^2^ for 4 h. **(A)** Expression of osteogenic genes (BMP2, RUNX2, ALP, and OCN) in MC3T3-E1 cells treated with conditioned medium for 12 h, assessed on days 7 and 14; **(B)** IF staining and fluorescence intensity of ALP and RUNX2 in MC3T3-E1 cells treated with conditioned medium for 12 h, assessed on days 7; **(C)** The ALP and ARS staining and fluorescence intensity in MC3T3-E1 cells treated with conditioned medium for 12 h, assessed on days 7, 14 and 21 days; **(D)** Schematic diagram of the static pressure model on Raw264.7 and the indirect co culture MC3T3 E1 model. (ns P ≥ 0.05, *P < 0.05, **P < 0.01).

These results indicate that Lira not only inhibits pressure-induced osteoclastogenesis but also indirectly promotes osteogenic differentiation by modulating the secretory function of macrophages. BMP2, RUNX2, ALP, and OCN are key markers of osteogenic differentiation, among which RUNX2 serves as the master transcription factor that directly activates downstream target genes including ALP and OCN, while BMP2 functions as an upstream signaling molecule that enhances RUNX2 transcriptional activity through the Smad pathway. Therefore, the upregulation of these osteogenic marker genes by Lira suggests that it may improve bone metabolic balance by enhancing the osteogenic supportive capacity of macrophages. Given that the present study has demonstrated that Lira suppresses the expression of M1-type inflammatory cytokines including TNF-α, iNOS, and IL-1β, it is plausible that Lira may indirectly enhance the osteogenic supportive capacity of macrophages by inhibiting their polarization toward the M1 phenotype. In the cell model of this study, considering that a certain amount of Lira may remain in the conditioned medium, the possibility that the osteogenic effect also includes a direct osteogenic-promoting effect of Lira cannot be excluded. This mechanism is consistent with previous reports demonstrating that GLP-1 receptor agonists can improve the bone metabolic microenvironment by modulating macrophage polarization states (He et al., 2025; Wang et al., 2017; Hou et al., 2020).

The osteogenic and osteoclastic markers examined in this study do not exist in isolation but rather reside within a mutually coupled signaling network. In the osteoclastic lineage, TRAF6, as a key adaptor protein in RANKL signaling, activates the downstream NF-κB pathway, driving NFATc1 expression and thereby activating osteoclastic functional genes such as TRAP; inflammatory factors such as TNF-α secreted by M1-polarized macrophages can further amplify this signal, forming a synergistic amplification loop between inflammation and osteoclastogenesis. In the osteogenic lineage, Lira promotes RUNX2 expression, which activates downstream osteogenic genes including ALP and OCN. Therefore, the downregulation of the TRAF6, NF-κB-NFATc1, TRAP and the upregulation of the BMP2, RUNX2, ALP/OCN observed in this study reflect the systemic regulation of the osteoclast-osteoblast coupling network by liraglutide. In summary, these results indicate that Lira exerts a positive regulatory effect on macrophage-osteoblast coupling under pressure conditions: on one hand, it indirectly promotes osteogenesis by suppressing inflammatory signals; on the other hand, it directly upregulates the expression of key osteogenic differentiation genes. This dual regulatory action—simultaneously inhibiting osteoclastogenesis while promoting osteogenesis—further supports the therapeutic potential of Lira in pressure-related bone metabolic disorders.This study indeed remains at the cellular level and has not yet been validated by animal experiments, which is one of the main limitations of this study. Although the cell model can, to a certain extent, reveal the regulatory effects of Lira on macrophage polarization and the osteogenic–osteoclastic balance under pressure conditions, the bone metabolic microenvironment *in vivo* involves multiple cell types, cytokine networks, and systemic regulatory factors, and its complexity cannot be fully reproduced by a single cell model. Therefore, in future work, we will strive to identify a suitable animal model that can better simulate abnormal mechanical force-induced joint inflammation, and on this basis systematically investigate the effects and underlying mechanisms of Lira in the *in vivo* setting. Through the mutual corroboration of cellular and animal experiments, we hope to provide more substantial experimental evidence for the clinical application of Lira in pressure-related bone disorders.

## 4 Conclusion

This study systematically investigated the effects of liraglutide on macrophage differentiation, inflammatory responses, and bone metabolic regulation under mechanical pressure. The results demonstrated that mechanical pressure directly drives macrophage differentiation toward osteoclasts through the NFATc1-TRAP signaling axis, while simultaneously inducing the release of inflammatory cytokines including TNF-α, iNOS, and IL-1β, thereby establishing a synergistic amplification between inflammatory and osteoclastogenic signals. Although liraglutide did not affect the expression of the upstream mechanosensor Piezo1, it significantly suppressed the mRNA and protein expression of NFATc1 and TRAP, and attenuated inflammatory responses by inhibiting the TRAF6 and NF-κB, thus disrupting the synergistic crosstalk between inflammation and osteoclastogenesis. Furthermore, liraglutide indirectly promoted osteogenic differentiation by modulating macrophage secretory function, as evidenced by significant upregulation of osteogenic marker genes including BMP2, RUNX2, ALP, and OCN in MC3T3-E1 cells. Collectively, liraglutide effectively reversed pressure-induced bone metabolic imbalance through its triple actions—inhibiting osteoclastogenesis, exerting anti-inflammatory effects, and indirectly promoting osteogenesis—thereby providing a theoretical basis and experimental evidence for its therapeutic potential in pressure-related degenerative joint diseases.

## Data Availability

The original contributions presented in the study are included in the article/Supplementary Material, further inquiries can be directed to the corresponding author/s.
