# Supplementary material for: Liraglutide regulate osteogenesis-resorption via macrophage under compressive stress
Source: Front Pharmacol. 2026 Oct 26;17:1985291. doi: 10.3389/fphar.2026.1985291 (PMC13745719; doi:10.3389/fphar.2026.1985291)
Supplement: Supplementary file 1 [file Supplementaryfile1.docx]

Supplementary Material

**Supplementary Material**

Graphical Abstract

Figure 1. The effects of compressive pressure on macrophage viability and function treated by compressive pres-sure. (A) The viability of Raw264.7; (B) The live-dead staining of Raw264.7; (C) The Piezo1 mRNA expression levels; (D) The NFATc1 mRNA expression levels; (E) The TRAP mRNA expression levels; (F) The TNF-α mRNA expres-sion levels; (G) The iNOS mRNA expression levels; (H) The IL-1β mRNA expression levels.

Figure 2. The effects of Lira on pressure‑induced osteoclastogenesis in Raw264.7. (A) Schematic diagram of Lira on macrophage differentiation mechanisms under mechanical pressure; (B) The Piezo1 mRNA expression levels; (C) The NFATc1 mRNA expression levels; (D) The TRAP mRNA expression levels; (E) IF staining of NFATc1 and TRAP; (F) NFATc1 and TRAP protein expression levels; (G) Schematic diagram of the static pressure model on macrophag-es and the indirect co‑culture MC3T3‑E1 model.

Figure 3. The effects of Lira on pressure‑induced inflammation in Raw264.7. (A) The expression of inflammatory cy-tokines (TNF‑α, iNOS, IL‑1β and NF‑κB); (B) IF staining of TRAF6 and p-P65; (C) TRAF6, P65 and p-P65 protein ex-pression levels.

Figure 4. The effects of Lira on pressure‑treated MC3T3-E1 differentiation via Raw264.7. (A) The expression of oste-ogenesis genes (BMP2, RUNX2, ALP, and OCN); (B) IF staining of ALP and RUNX2.

Table 1. Primer sequences for qRT‑PCR

| Gene | Forward primer (5'‑3') | Reverse primer (5'‑3') |
| --- | --- | --- |
| GAPDH | TGTGTCCGTCGTGGATCTGA | TTGCTGTTGAAGTCGCAGGAG |
| piezo1 | GCAGCCGAGAGACAGAGAAG | AGAGCAGAGGGAACCAGATGA |
| NFATc1 | AGAAGATGGTGCTGTCTGGC | TCCCGGTCAGTCTTTGCTTC |
| TNF-α | CAGAAAGCATGATCCGCGAC | CTGCCACAAGCAGGAATGAG |
| iNOS | ACGAGACGGATAGGCAGAGA | GCACATGCAAGGAAGGGAAC |
| IL-1β | TGGTGTGTGACGTTCCCATTA | TCGTTGCTTGGTTCTCCTTGT |
| NF-κB | AGGCAAGGAATAATGCTGTCCTG | ATCATTCTCTAGTGTCTGGTTGG |
| TRAP | TGGTCATTTCTTTGGGGCTTATCT | GCTACTTGCGGTTTCACTATGGA |
| ALP | TACATTCCCCATGTGATGGC | AGAGCAGAGGGAACCAGATGA |
| RUNX2 | TCTCCAACCCACGAATGCACTA | ATAGCGTGCTGCCATTCGAGGT |
| BMP-2 | AACGAGAAAAGCGTCAAGCC | AGGTGCCACGATCCAGTCAT |
| OCN | CAGAACAGCAAGTCCCACACAG | TCAGCAGAGTGAGCAGAAAGAT |
